# High Prevalence of *Mycoplasma penetrans* in *Chlamydia trachomatis* Positive Rectal Samples From Men: A Brief Report

**DOI:** 10.3389/fmicb.2022.914874

**Published:** 2022-06-13

**Authors:** Inmaculada Pérez-Prieto, Axel Skafte-Holm, Jørgen Skov Jensen

**Affiliations:** ^1^Department of Biochemistry and Molecular Biology, Faculty of Sciences, University of Granada, Granada, Spain; ^2^Instituto de Investigación Biosanitaria ibs.GRANADA, Granada, Spain; ^3^Department of Bacteria, Parasites and Fungi, Research Unit for Reproductive Microbiology, Statens Serum Institut, Copenhagen, Denmark

**Keywords:** *Mycoplasma penetrans*, *Chlamydia trachomatis*, coinfection, sexually transmitted infections, rectum

## Abstract

*Mycoplasma penetrans* has gained increased attention in relation to sexually transmitted infections, however, its pathogenic potential and prevalence in different populations remains to be elucidated. Among 293 *Chlamydia trachomatis* positive rectal samples submitted for lymphogranuloma venereum typing, *M. penetrans* was detected by PCR in 13.4% of 231 male samples.

## Introduction

Sexually transmitted infections (STIs) are caused by a wide range of bacteria, viruses and parasites. According to WHO estimates, more than 1 million STIs occur every day, emerging as an increasing reproductive and sexual health concern worldwide (World Health Organization, 2019). In this context, a high prevalence of STIs among men who have sex with men (MSM) is reported, with *Chlamydia trachomatis* and *Neisseria gonorrhoeae* being the most important (Workowski et al., 2021). Serotype L1-L3 of *C. trachomatis* causes lymphogranuloma venereum (LGV), a destructive and aggressive sexually transmitted infection affecting tissue and lymph nodes, predominantly associated with rectal infection in MSM in developed countries (Stoner and Cohen, 2015). Thus, genotyping of *C. trachomatis* positive rectal samples has been recommended since these infections require extended duration of therapy (De Vries et al., 2019).

A significant proportion of patients with STI symptoms have no identified etiological agents detected. Advances in molecular methods have allowed the search for unidentified pathogens by non-culture-dependent techniques. In this regard, several studies have reported the presence of *Mycoplasma* spp., primarily *M. genitalium*, in conditions such as non-gonococcal urethritis (NGU) (Jensen et al., 1993; Taylor-Robinson et al., 2003). In addition, a recent publication found *M. penetrans* to be associated with NGU primarily in MSM (Srinivasan et al., 2021). Since anal sex increases the risk for extra-genital infections, we assessed the prevalence of *M. penetrans* in *C. trachomatis* positive rectal samples submitted for LGV typing.

## Materials and Methods

### Study Setting

*Chlamydia trachomatis* positive samples from diagnostic laboratories throughout Denmark between March and October 2021 were submitted for molecular LGV typing in the reference laboratory at Statens Serum Institut. Of the 837 original samples, remnants of 293 rectal samples were available and anonymized to gender and age, and analyzed for *M. penetrans*. According to Danish law, an ethical committee approval was not required due to the anonymized nature of the dataset.

### Sample Preparation

DNA was extracted from the samples submitted in the nucleic acid amplification test transport medium by processing 1 ml of sample using the Large Volume Universal Pathogen Extraction protocol in a MagNA Pure 96 instrument (Roche Diagnostics, Hvidovre, Denmark) and was eluted into 100 μl.

### Real-Time PCR (qPCR)

*Mycoplasma penetrans* was detected by qPCR as previously described (Srinivasan et al., 2021), except that an internal process control (IPC) was constructed in order to detect Taq DNA polymerase inhibitors or suboptimal reaction conditions (Jensen et al., 2003). Each assay was performed in a 50 μl final reaction volume in a 7,500 Real-Time PCR System instrument (Thermo Fisher Scientific, Waltham, MA, United States), programmed for 95°C for 2 min and 50 cycles of 95°C for 15 s and annealing and extension at 60°C for 1 min. Standard curves were prepared with serial dilutions of *M. penetrans* DNA containing 50,000 to 5 copies.

## Results

Results of *M. penetrans* detection are summarized in Table 1. A total of 293 rectal samples (231 of male and 62 of female origin) were included in this study. The median age was 30 years ranging from 16 to 69. Overall, *M. penetrans* was detected more frequently in male *C. trachomatis* positive samples with a prevalence of 13.4% (95%CI 9.30–18.5) compared to 1.6% (95%CI 0.00–8.66), in female samples (*p* = 0.005, Fisher’s exact test). *M. penetrans* DNA concentrations ranged from 2 to 31,846 genome equivalents (GEQ)/μl with a median of 33 GEQ/μl.

**TABLE 1:** Distribution of *M. penetrans* among *C. trachomatis* positive rectal samples.

Gender	Rectal samples, n	*M. penetrans* detected, n	Percentage (95% CI)
Male	231	31	13.4 (9.3–18.5)
Female	62	1	1.6 (0.0–8.7)

## Discussion

In this report, we describe a high prevalence of *M. penetrans* in male *C. trachomatis* positive rectal samples, in accordance to previous results (Taylor-Robinson et al., 2003). Earlier studies have mainly aimed to detect *M. penetrans* in male populations, including healthy MSM and men who have sex with women (MSW). In the 1990’s, shortly after the discovery of *M. penetrans*, several publications highlighted its potential role in the progression of HIV infection to AIDS and for development of Kaposi’s sarcoma by analyzing *M. penetrans* antibodies in serum samples from healthy controls and patients of different risk groups for AIDS (Wang et al., 1992, 1993). In our study, lack of clinical information prevented us to evaluate the association between *M. penetrans* and HIV positivity.

Two studies have detected *M. penetrans* by PCR with a prevalence of 1.4 and 1.6%, respectively, in urine samples from HIV-infected individuals (Wang et al., 2012; Chen et al., 2015). One of these studies also included sexually transmitted clinic attendees and healthy controls, where the *M. penetrans* infection rate was < 1% (Wang et al., 2012). An earlier study of urethral swabs from Japanese men with and without NGU failed to detect *M. penetrans* in any of the patients (Deguchi et al., 1996). *M. penetrans* has been detected in rectal samples from 28 MSM with or without NGU with 10 vs. 5.6% positives, respectively. In addition, the study found a 10% prevalence of *M. penetrans* in urethral and throat samples in the men with NGU (Taylor-Robinson et al., 2003). Recent evidence has suggested a role of *M. penetrans* in NGU, a syndrome with unknown etiology in > 50% of cases. In search of an etiology for idiopathic NGU, Srinivasan et al. found an association between detection of *M. penetrans* and NGU by 16S microbiota analysis. They confirmed the findings using qPCR in urine samples from 431 men with and without NGU. *M. penetrans* was found in 8% of men diagnosed with NGU compared to 1% in men without NGU (Srinivasan et al., 2021). However, the association was found only in MSM where 13% of the NGU cases were *M. penetrans* positive compared to 3% of MSW with NGU. The present study revealed a 13% prevalence of *M. penetrans* in male *C. trachomatis* positive rectal samples. Due to the anonymization, we are not able to determine the proportion of MSM in our population, but it is probably nearly 100%, as rectal swabs are rarely obtained from MSW. Among MSM, bacterial STIs are common in the rectal site, and some studies have found a much higher positive rate in rectal swabs as compared to urine (Munson et al., 2021).

Due to the absence of cell wall, *Mycoplasma* spp. are naturally resistant to a wide spectrum of antibiotics. The most frequently used treatment of infection are macrolides, tetracyclines and fluoroquinolones, however, in the urogenital pathogen *M. genitalium*, several mutations associated with macrolide and fluoroquinolone resistance have been identified (Machalek et al., 2020). Only few isolates of *M. penetrans* are available and the literature is sparse on its antimicrobial susceptibility, however, early studies suggested that most isolates of *M. penetrans* were susceptible to all three antimicrobial classes (Hayes et al., 1995). In a more recent study, one *M. penetrans* strain showed evidence of macrolide resistance (Duffy et al., 2000). Similarly, a recent communication found complete resistance to azithromycin on four urogenital isolates from immunocompetent men suffering idiopathic NGU (Schwab et al., 2021). This development suggests that macrolide resistance in *M. penetrans* may be increasing over time, just as it has been shown for *M. genitalium* (Machalek et al., 2020). Altogether, these results emphasize the need for collection of contemporary *M. penetrans* isolates with antimicrobial susceptibility testing and molecular studies to determine the basis for the resistance in this emerging etiologic agent of NGU.

There are several limitations to our study. Due to the anonymized nature of the samples, important clinical, demographic and behavioral data are missing. Furthermore, it was not possible to evaluate whether multiple samples from the same subject were included, which could affect numbers. The high prevalence of *M. penetrans* in male rectal swabs supports the findings from previous studies of a strong link between MSM behavioral factors and *M. penetrans* detection. Future research should evaluate the role of *M. penetrans* as a pathogen potentially implicated in different STIs but should emphasize differences in sexual behavior.

## Conclusion

The prevalence of *M. penetrans* in *C. trachomatis* positive male rectal swabs was surprisingly high at 13%. Although clinical data were missing because of anonymization, the presence of *M. penetrans* appears to be strongly associated with MSM behavior as suggested also in previous studies. Our findings indicate that *M. penetrans* may cause coinfection with *C. trachomatis*, however, the implications for symptoms and treatment outcomes require further research.

## Data Availability Statement

The original contributions presented in the study are included in the article/supplementary material, further inquiries can be directed to the corresponding author.

## Ethics Statement

Ethical review and approval was not required for the study on human participants in accordance with the Local Legislation and Institutional Requirements. Written informed consent for participation was not required for this study in accordance with the National Legislation and the Institutional Requirements.

## Author Contributions

IP-P performed the experimental assays and wrote the manuscript. AS-H participated in writing phase and statistical analysis, elaborated the table, and reviewed the initial manuscript draft for important intellectual content. JSJ conceptualized and designed the study, supervised the manuscript, and reviewed the manuscript for important intellectual content. All authors contributed to the article and approved the submitted version.

## Conflict of Interest

JSJ reports grants, personal fees, and non-financial support from Hologic, personal fees from Roche, grants and personal fees from SpeeDx, grants and personal fees from Nabriva, grants and personal fees from Cepheid, grants and personal fees from Abbott, and grants and personal fees from GSK all outside the submitted work. The remaining authors declare that the research was conducted in the absence of any commercial or financial relationships that could be construed as a potential conflict of interest.

## Publisher’s Note

All claims expressed in this article are solely those of the authors and do not necessarily represent those of their affiliated organizations, or those of the publisher, the editors and the reviewers. Any product that may be evaluated in this article, or claim that may be made by its manufacturer, is not guaranteed or endorsed by the publisher.
